# Analysis of Postoperative Outcomes in Unilateral Versus Bilateral DIEP Flap Reconstructions: A Single-Center Retrospective Study

**DOI:** 10.3390/jcm14041056

**Published:** 2025-02-07

**Authors:** Boran Tekdogan, Jérôme Martineau, Miroslava Verbat, Edward T. C. Dong, Daniel Correia, Carlo M. Oranges

**Affiliations:** 1 Department of Plastic, Reconstructive, and Aesthetic Surgery, Geneva University Hospitals, Geneva University, 1205 Geneva, Switzerland; boran-can.tekdogan@hug.ch (B.T.); jerome.martineau@hug.ch (J.M.); edward.dong@hug.ch (E.T.C.D.); daniel.correia@hug.ch (D.C.); 2 First Faculty of Medicine, Charles University in Prague, Kateřinská 1660/32, 121 08 Prague, Czech Republic; 10820786@cuni.cz

**Keywords:** DIEP flap, breast reconstruction, unilateral reconstruction, bilateral reconstruction, complication rates, surgical outcomes, reconstructive surgery, autologous breast reconstruction

## Abstract

**Background/Objectives**: The DIEP flap is among the preferred techniques in autologous breast reconstruction due to better long-term outcomes, including higher satisfaction and more natural breast shape compared to implant-based breast reconstruction. With the rise in genetic testing, bilateral DIEP reconstructions are becoming more common, though they carry a higher risk of complications. This study aims to compare the risks between unilateral and bilateral procedures to improve surgical decision-making. **Methods**: A retrospective, single-center review was conducted on female patients who underwent DIEP flap breast reconstruction between January 2018 and May 2024. The study included patients with complete medical records and follow-up data, excluding those with incomplete records. Patient characteristics, operative details, and complications were thoroughly analyzed, with donor site complications assessed per patient and recipient site complications per breast. **Results**: During the study, 141 DIEP flaps were performed on 114 women, with 87 unilateral and 27 bilateral reconstructions. Age and BMI were similar between groups. However, chemotherapy was more common in the bilateral group (85% vs. 47%, *p* = 0.0011). Operative time was significantly longer in bilateral procedures (650 vs. 460 min, *p* < 0.0001). There were no statistically significant differences in recipient and donor site complications across groups. The hospital stay was significantly longer in the bilateral group (11 vs. 8.8 days, *p* = 0.024). **Conclusions**: Bilateral and unilateral DIEP flap breast reconstructions have similar complication and early take-back rates.

## 1. Introduction

Autologous breast reconstructions, particularly those using the deep inferior epigastric artery perforator (DIEP) flap, are increasingly favored over implant-based reconstructions due to several significant advantages. Many studies demonstrated that patients undergoing DIEP report better long-term satisfaction, superior quality of life, and obtain a more natural aesthetic outcome compared to those opting for implant-based reconstructions [1,2,3,4,5,6,7,8,9,10,11,12,13,14].

Advances in genetic testing, such BRCA1 and BRCA2 mutations screening, have led to a rise in bilateral prophylactic mastectomies. These surgeries are increasingly chosen to reduce the risk of breast cancer through early identification of genetic risks [15,16,17,18,19,20,21,22]. Consequently, bilateral DIEP flap reconstructions are becoming more common, as they provide a natural and durable solution to restore breast symmetry and aesthetics following prophylactic mastectomy.

Although bilateral reconstructions offer better symmetry and aesthetic outcomes, studies comparing postoperative complications between unilateral and bilateral DIEP reconstructions remain limited. A significant meta-analysis by Wormald et al., published in 2014, showed that bilateral reconstructions were associated with a significantly higher risk of total flap failure [23]. These results do not appear to reflect the experience at our center, and this study aims to compare postoperative complications following unilateral and bilateral DIEP flap reconstruction to better guide our patients in their decision-making.

## 2. Materials and Methods

A retrospective review of consecutive female patients who underwent DIEP flap breast reconstruction at a single center between January 2018 and May 2024 was performed. Inclusion in the study was based on the availability of complete medical records and postoperative follow-up data. Patients with incomplete medical records were excluded.

Informed consent was obtained from all patients, and the study received approval from the competent institutional review board (project ID: CCER 2024-01103).

Patient characteristics and risk factors were evaluated, including the date of operation, type of reconstruction (unilateral or bilateral), timing of reconstruction (primary or secondary), operative details, length of hospital stay, follow-up duration, and any postoperative complications, including surgical re-explorations. Donor site complications were analyzed on a per-patient basis, while recipient site complications were assessed on a per-breast basis.

The data analysis was performed using R software version 4.2.1 (R Foundation for Statistical Computing, Vienna, Austria). Patient groups were compared using independent two-sided *t*-tests for means, Mann–Whitney U tests for comparing distributions (medians), and two-sided chi-square or Fisher’s exact test, as appropriate, to analyze categorical variables. Fisher’s exact test was used specifically when sample sizes were small or when the assumptions for the chi-square test were not met. The normality of continuous variables was assumed based on the data distribution, and non-parametric tests were used where appropriate. Statistical significance was set at a *p* value < 0.05.

## 3. Results

**Figure 1:**
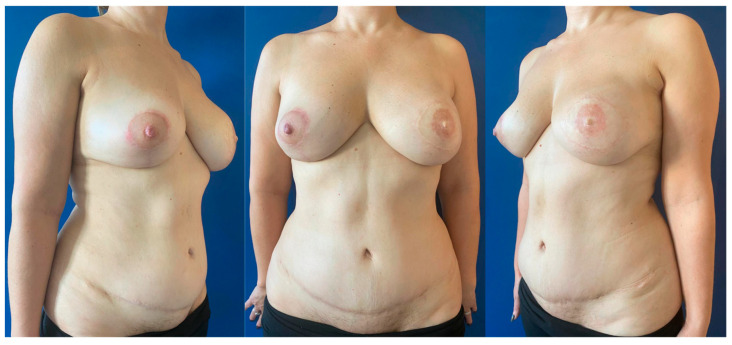
Postoperative views of a delayed unilateral left breast reconstruction using a DIEP flap at 16 months follow-up. The patient previously underwent a unilateral skin-sparing mastectomy of the left breast and immediate implant-based reconstruction. In the delayed procedure, the implant was removed and the unilateral autologous reconstruction was performed. Later, the patient underwent symmetrization mastopexy of the right breast with a superior pedicle and simultaneous nipple–areola complex reconstruction of the left breast using a full-thickness skin graft harvested from the medial aspect of the right thigh for the areola and an arrow flap for the nipple.

**Figure 2:**
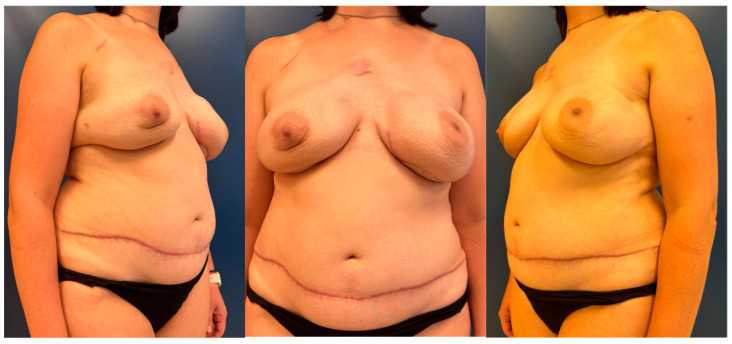
Postoperative views of a delayed bilateral breast reconstruction using a double DIEP flap at 4.5 months follow-up. The patient previously underwent a bilateral nipple-sparing mastectomy (prophylactic on the right) with immediate implant-based reconstruction. In the delayed procedure, the implants were removed and the bilateral autologous reconstruction was performed. The monitoring skin paddles are still in place, and their removal is planned.

During the study period, 114 women underwent a DIEP flap breast reconstruction, including 87 unilateral reconstructions (Figure 1) and 27 bilateral reconstructions (Figure 2), representing a total of 141 DIEP flaps. All DIEP breast reconstructions, either unilateral or bilateral, were carried out in a university setting by a microsurgical team always including at least one experienced microsurgeon.

The mean age was 51 years (SD 9.3) in the unilateral group and 48 years (SD 7.7) in the bilateral group, with a trend towards a younger age in the bilateral group (*p* = 0.11). The mean BMI was 26 kg/m^2^ (SD 4.3) in the unilateral group and 28 (SD 4.2) in the bilateral group (*p* = 0.22). There was no significant difference between the two groups regarding the proportion of smokers, hypertension, diabetes, and the presence of abdominal scars. There was no significant difference in the history of adjuvant radiotherapy between the two groups. However, a significantly higher proportion of patients in the bilateral group received chemotherapy (85% vs. 47%, *p* = 0.0011). The mean follow-up was 17 months (SD 15) in the unilateral group and 18 months (SD 14) in the bilateral group, with no significant difference between the groups (*p* = 0.64) (Table 1).

Curative procedures were significantly more frequent in the unilateral group (95% vs. 41%, *p* < 0.0001), while prophylactic procedures were more common in the bilateral group (59% vs. 4.6%, *p* < 0.0001). All reconstructions were performed using the internal mammary vessels as the recipient site. The mean operation time was significantly longer in the bilateral group, with an average of 650 min (SD 160) compared to 460 min (SD 85) in the unilateral group (*p* < 0.0001). However, the mean ischemia time per breast was similar between the two groups (Table 2).

Recipient site complications were similar between the two groups. In our series, one patient in the unilateral group (1.1%) and one patient in the bilateral group (1.9%) had a total flap loss, with no significant difference across groups. Additionally, partial flap loss occurred in 1.1% of the unilateral cases compared to 3.7% of the bilateral cases, with no statistically significant difference across groups. Early take-back procedures were necessary for six patients (6.9%) in the unilateral group and two patients (7.4%) in the bilateral group. In the unilateral group, interventions included surgical hematoma evacuation for two patients, a return to the operating room for hemostasis in one patient due to diffuse bleeding, flap revision due to venous congestion in another, re-anastomosis for a venous thrombosis in one patient, and re-anastomosis for arterial thrombosis on postoperative day 3 in another patient. Unfortunately, the flap revision for the patient with arterial thrombosis was unsuccessful, leading to a flap loss. In the bilateral group, take-back operations included surgical hematoma evacuation for one patient and re-anastomosis for arterial thrombosis in another patient, with successful salvage, despite a partial flap loss. At least one recipient site complication was reported in 18% of unilateral cases and 17% of bilateral cases. Seromas and wound infections were rare, with only one case each in the unilateral group. Hematomas occurred in 4.6% of unilateral cases and 3.7% of bilateral cases. Wound dehiscence and delayed wound healing were slightly more frequent in the unilateral group (4.6%) compared to 1.9% in the bilateral group. Partial NAC necrosis occurred in one case in both groups. None of these differences were statistically significant (Table 3).

Donor site complications were largely similar between the two groups. Wound dehiscence occurred in 8.0% of the unilateral cases and 11% of the bilateral cases, with no significant difference. Delayed wound healing was observed in 9.2% of patients in the unilateral group and 15% of patients in the bilateral group (*p* = 0.47). Seromas were more frequent in the unilateral group (6.9%) compared to the bilateral group (0%), although this difference was not statistically significant. Hematomas were observed at similar rates in both groups, with 3.4% in the unilateral group and 3.7% in the bilateral group, and this difference was also not statistically significant. Wound infections were slightly more common in the bilateral group (3.7% vs. 1.1%), albeit not statistically significant. Incisional hernias were rare, occurring in only one case in the unilateral group (1.1%) and none in the bilateral group (*p* = 1.0). Hypertrophic scars were observed in 2.3% of unilateral cases and 3.7% of bilateral cases, with no significant difference between the groups. Dog-ear scar deformities were slightly more frequent in the bilateral group (7.4% vs. 3.4% for the unilateral group), but this difference was also not statistically significant. Overall, at least one donor site complication was reported in 33% of unilateral cases and 41% of bilateral cases, with no significant difference between the groups (Table 4). The mean hospital stay was significantly longer in the bilateral group, with an average of 11 days (SD 5.0) compared to 8.8 days (SD 2.4) in the unilateral group (*p* = 0.024).

## 4. Discussion

Breast reconstruction using the DIEP flap has become one of the preferred techniques for post-mastectomy surgeries, offering high patient satisfaction and safety [2,24,25,26]. While complications associated with this method are well understood, literature on the specific differences in postoperative outcomes between unilateral and bilateral reconstructions is scarce. As bilateral procedures are becoming increasingly common, particularly among patients undergoing prophylactic mastectomies, understanding these differences is essential to optimize patient care, guide surgical decisions, and provide personalized risk to patients considering bilateral versus unilateral reconstruction [27].

Our study revealed that the demographic characteristics of patients were generally similar between the unilateral (UL) and bilateral (BL) reconstruction groups, with comparable profiles in terms of BMI, smoking status, and comorbidities such as hypertension and diabetes. However, we observed a trend towards a slightly lower average age in the BL reconstruction group, although this difference was not statistically significant. This trend may be partially explained by the fact that BL reconstructions are often chosen by patients with genetic or familial risk factors, such as BRCA1 or BRCA2 mutations, which are linked to an earlier onset of breast cancer [16,28,29,30].

However, the rate of radiotherapy before reconstruction was higher in the BL reconstruction group, although this difference was not statistically significant (*p* = 0.083). This suggests a potential for increased complications in the BL group, as radiotherapy has been associated with higher risks of recipient site complications [31,32].

We did not observe any significant difference in recipient site or donor site postoperative complications between UL and BL reconstructions. Rates of partial or total flap loss were similar between the groups, with one total flap loss in the unilateral group and one in the bilateral group. The rate of partial flap loss was also comparable, with one case in the unilateral group and two cases in the bilateral group. To compare the results of our study with the current literature, we refer to Wormald’s meta-analysis, which combined data from 17 studies, representing a total of 2398 patients [23]. Their analysis showed that bilateral DIEP reconstructions were associated with a significantly higher risk of total flap loss which does not align with our findings. A recent meta-analysis by Tekdogan et al. showed that while there was a significantly higher risk of complete flap loss following bilateral cases, the increase in risk was moderate and lesser than what Wormald et al. reported [33]. These differences may be explained by the fact that total flap loss is a rare complication, as it has become a routine procedure. Moreover, our study included only a limited number of patients. However, a recent study by Moellhoff et al., also comparing unilateral and bilateral DIEP reconstructions, did not find any significant differences in the rate of total flap loss, despite a larger sample size of 3926 patients, thus supporting the findings of our study [34].

One of the interesting points of the current study is that the length of hospital stay was significantly longer in the BL group compared to the UL group by more than 2 days. This increase may be attributed to the greater operative complexity of BL reconstructions, requiring extended postoperative monitoring, more intensive pain management, and mobility limitations, thus prolonging the need for hospital care even without additional complications. These results contrast with the study by Moellhoff et al., where a shorter hospital stay in the BL group was observed [34]. The authors explained this difference by the significantly lower age in the BL group. Although our study also showed a trend towards a lower average age in the BL group, this difference was not statistically significant, which may explain the divergence in the observed lengths of stay.

Our study has some limitations that should be acknowledged. Firstly, the small sample size, especially for the bilateral group, limits the ability to detect statistically significant differences for rare events, such as total flap loss. Secondly, the retrospective nature of the study relies on existing medical records, which may lead to incomplete data and potential biases. To improve the study, future research should aim for a larger sample size and adopt a prospective design, allowing for better control of confounding factors and more robust data collection, enhancing the validity of the findings.

## 5. Conclusions

In conclusion, bilateral DIEP flap breast reconstructions appear to be performed with complication and re-exploration rates comparable to those of unilateral reconstructions. However, due to the limited size of this study, further research with larger patient cohorts is needed to validate these findings and more thoroughly assess the safety and outcomes of bilateral procedures.

## Figures and Tables

**Table 1:** Patient baseline characteristics.

	Unilateral (*n* = 87)	Bilateral (*n* = 27)	*p*-Value
Age [years], mean (SD)	51 (9.3)	48 (7.7)	0.11
BMI [kg/m^2^] (SD)	26 (4.3)	28 (4.2)	0.22
Smoker, *n* (%)	21 (24%)	7 (26%)	0.76
Hypertension, *n* (%)	14 (16%)	3 (11%)	0.76
Diabetes, *n* (%)	1 (1.1%)	0 (0%)	>0.99
Abdominal Scar, *n* (%)	46 (53%)	12 (44%)	0.59
Radiotherapy, *n* (%)	30 (34%)	15 (56%)	0.083
Chemotherapy, *n* (%)	41 (47%)	23 (85%)	0.0011
Hormone Therapy, *n* (%)	48 (55%)	14 (52%)	0.94
Follow-up [months], mean (SD)	17 (15)	18 (14)	0.64

**Table 2:** Operative characteristics.

	Unilateral (*n* = 87)	Bilateral (*n* = 27)	*p*-Value
Primary reconstruction, *n* (%)	11 (13%)	7 (26%)	0.13
Secondary reconstruction, *n* (%)	76 (87%)	20 (74%)	0.13
Curative, *n* (%)	83 (95%)	11 (41%)	<0.0001
Prophylactic, *n* (%)	4 (4.6%)	16 (59%)	<0.0001
Mean operation time [minutes], mean (SD)	460 (85)	650 (160)	<0.0001
Mean ischemia time (SD) [minutes], per breast	95 (33)	92 (34)	0.69

**Table 3:** Recipient site complications.

	Unilateral (*n* = 87)	Bilateral (*n* = 54)	*p*-Value
Breast recipient site			
≥1 complication, *n* (%)	16 (18%)	9 (17%)	0.79
Seroma, *n* (%)	1 (1.1%)	0 (0%)	>0.99
Hematoma, *n* (%)	4 (4.6%)	2 (3.7%)	>0.99
Wound infection, *n* (%)	1 (1.1%)	1 (1.9%)	>0.99
Wound dehiscence, *n* (%)	4 (4.6%)	1 (1.9%)	0.65
Delayed wound healing, *n* (%)	4 (4.6%)	1 (1.9%)	0.65
Partial NAC necrosis, *n* (%)	1 (1.1%)	1 (1.9%)	>0.99
DIEP flap			
Venous congestion, *n* (%)	1 (1.1%)	1 (1.9%)	>0.99
Venous thrombosis, *n* (%)	1 (1.1%)	0 (0%)	>0.99
Arterial thrombosis, *n* (%)	1 (1.1%)	1 (1.9%)	>0.99
Total flap loss, *n* (%)	1 (1.1%)	1 (1.9%)	>0.99
Partial flap loss, *n* (%)	1 (1.1%)	2 (3.7%)	>0.99

**Table 4:** Donor site complications.

	Unilateral (*n* = 87)	Bilateral (*n* = 27)	*p*-Value
≥1 complication, *n* (%)	29 (33%)	11 (50%)	0.48
Seroma, *n* (%)	6 (6.9%)	0 (0%)	0.33
Hematoma, *n* (%)	3 (3.4%)	1 (3.7%)	>0.99
Wound infection, *n* (%)	1 (1.1%)	1 (3.7%)	0.42
Wound dehiscence, *n* (%)	7 (8.0%)	3 (11%)	0.70
Delayed wound healing, *n* (%)	8 (9.2%)	4 (15%)	0.47
Hypertrophic scar, *n* (%)	2 (2.3%)	1 (3.7%)	0.56
Dog-ear scar deformity, *n* (%)	3 (3.4%)	2 (7.4%)	0.59
Incisionial hernia, *n* (%)	1 (1.1%)	0 (0%)	>0.99

## Data Availability

The data presented in this study are available on request from the corresponding author.
